# Supplementary material for: Transcriptome Analysis Reveals Vimentin-Induced Disruption of Cell–Cell Associations Augments Breast Cancer Cell Migration
Source: Cells. 2022 Dec 13;11(24):4035. doi: 10.3390/cells11244035 (PMC9776984; doi:10.3390/cells11244035)
Supplement: Supplementary file 1 [file cells-11-04035-s001.zip › DEGs and enrichment analysis.zip!/DEGs and enrichment analysis/WT_VIMvsVector GO enrichment/DOWN/WT_VIMvsVector_down.MFbar.pdf]

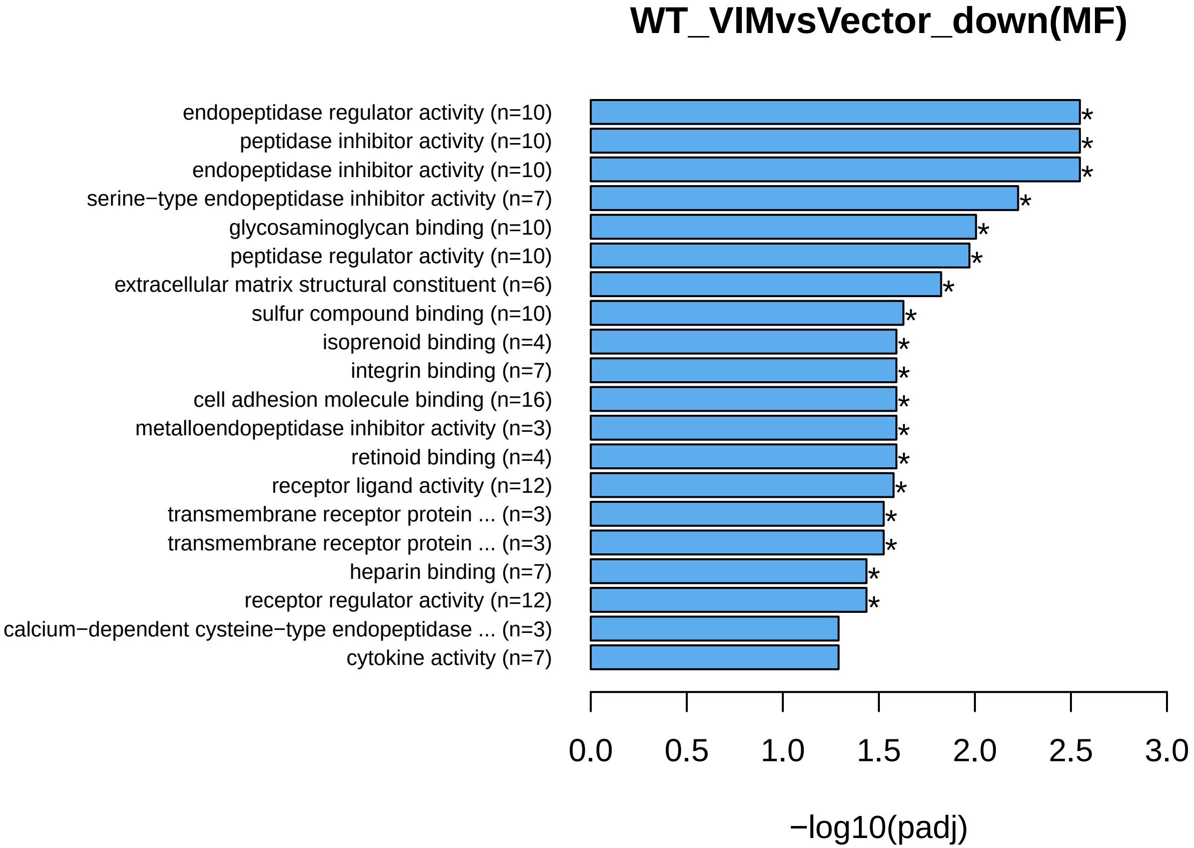
WT_VIMvsVector_down(MF)
endopeptidase regulator activity (n=10)
peptidase inhibitor activity (n=10)
endopeptidase inhibitor activity (n=10)
serine−type endopeptidase inhibitor activity (n=7)
glycosaminoglycan binding (n=10)
peptidase regulator activity (n=10)
extracellular matrix structural constituent (n=6)
sulfur compound binding (n=10)
isoprenoid binding (n=4)
integrin binding (n=7)
cell adhesion molecule binding (n=16)
metalloendopeptidase inhibitor activity (n=3)
retinoid binding (n=4)
receptor ligand activity (n=12)
transmembrane receptor protein ... (n=3)
transmembrane receptor protein ... (n=3)
heparin binding (n=7)
receptor regulator activity (n=12)
calcium−dependent cysteine−type endopeptidase ... (n=3)
cytokine activity (n=7)
0.0
0.5
1.0
1.5
2.0
2.5
3.0
−log10(padj)
